# Predicting Spatial Visualization Problems’ Difficulty Level from Eye-Tracking Data

**DOI:** 10.3390/s20071949

**Published:** 2020-03-31

**Authors:** Xiang Li, Rabih Younes, Diana Bairaktarova, Qi Guo

**Affiliations:** 1 School of Electrical and Electronic Engineering, Huazhong University of Science and Technology, Wuhan 430074, China; xiangli_ee@hust.edu.cn; 2 Department of Electrical and Computer Engineering, Duke University, Durham, NC 27708, USA; 3 Department of Engineering Education, Virginia Tech, Blacksburg, VA 24061, USA; dibairak@vt.edu; 4 International School, Beijing University of Posts and Telecommunications, Beijing 100876, China; guoqi@bupt.edu.cn

**Keywords:** eye-tracking, spatial visualization, machine learning, proactive systems, engineering education

## Abstract

The difficulty level of learning tasks is a concern that often needs to be considered in the teaching process. Teachers usually dynamically adjust the difficulty of exercises according to the prior knowledge and abilities of students to achieve better teaching results. In e-learning, because there is no teacher involvement, it often happens that the difficulty of the tasks is beyond the ability of the students. In attempts to solve this problem, several researchers investigated the problem-solving process by using eye-tracking data. However, although most e-learning exercises use the form of filling in blanks and choosing questions, in previous works, research focused on building cognitive models from eye-tracking data collected from flexible problem forms, which may lead to impractical results. In this paper, we build models to predict the difficulty level of spatial visualization problems from eye-tracking data collected from multiple-choice questions. We use eye-tracking and machine learning to investigate (1) the difference of eye movement among questions from different difficulty levels and (2) the possibility of predicting the difficulty level of problems from eye-tracking data. Our models resulted in an average accuracy of 87.60% on eye-tracking data of questions that the classifier has seen before and an average of 72.87% on questions that the classifier has not yet seen. The results confirmed that eye movement, especially fixation duration, contains essential information on the difficulty of the questions and it is sufficient to build machine-learning-based models to predict difficulty level.

## 1. Introduction

The quality of teaching has made a huge leap with the help of digital technology. Among these technologies, multimodal learning analytics (MMLA) combines data from multiple sources for analysis and processing, to achieve the purpose of multi-dimensional analysis of the learning process. In the well-established field of multimodal fusion, data collection and sensing technologies make it possible to collect sufficient and reliable human activity data to support MMLA. Technologies currently widely used in human activity data collection include: wearable cameras, wearable sensors, biosensors, infrared imaging, and eye-tracking [1].

In MMLA, eye-tracking technology serves as an important tool to help researchers and machines understand human thoughts and behaviors [1]. Its reliability and universality allow it to be widely used in building real-time interfaces in human-computer interaction (HCI) research and in investigating human mental activity. The eye-tracking data could be used to reconstruct the human thinking process in a real-time, consecutive and sensitive way and also offers an opportunity to the researcher to better understand human mental and physical behaviors. Based on eye-tracking technology, some models had been developed to automatically detect human behaviors.

Furthermore, several former studies—e.g., [2,3]—indicate that eye movement during the observation of complex visual stimuli is systematic and ordered, which shows the possibility for building cognitive and perceptual processes model based on eye-tracking data. For example, differences in the difficulty of questions on a test are often thought to be reflected in the characteristics of eye-tracking data [4]. These differences make it possible to detect the mental effort and track emotional changes during a test.

Recent investigations of eye movement and cognitive processes are almost based on an eye-mind hypothesis (i.e., inferring the cognitive activity reflected by the accurate description of eye movement) [5]. Eye-tracking data is also further explored and was found to contain important information on visual attention [6], mental effort, and cognitive load [5]. To investigate the usability of eye-tracking from the perspective of practical applications, studies in reading comprehension, visual search, selective attention, and studies of visual working memory [7] have been performed. Based on this information, research yielded several models in the human cognition field. Conati and Mertena [8] pointed out that eye-tracking data helps probabilistic models have a better performance in online assessment tasks. Eivazi and Bednarik [9] built a general model to recognize cognitive states. They argued that eye-tracking data could be used to build a user cognition states model which could contribute to real-time learning process evaluation.

Concerning eye-tracking during the learning process, a work by Velichkovsky et al. [10] states that eye retention time increases with the difficulty of the cognitive process. In other words, when people are faced with simple tasks, their retention time is short. Long retention time indicates that they are dealing with more difficult cognitive processes [11]. Chen et al. [4] indicated that dwelling time in different areas of interest (AOIs) shows a big difference among questions in different difficulty levels. The difference in the eye-movement pattern makes it possible to predict difficulty level from eye-tracking data.

Our long-term goal is to build a proactive tutoring system that enables computers to evaluate the user’s mental effort and adjust the difficulty of questions automatically. As with many previous studies, eye-tracking data has the potential to be used to build cognitive models for problem-solving.

In this paper, first, we investigate the difference of eye-movement patterns when participants are solving spatial visualization problems with different difficulty levels. Second, we build SVM-based models to classify the difficulty level using eye-tracking data. Both question-dependent and question-independent tests are conducted to show the capability of our classifiers in predicting difficulty level. The potential application of the question-dependent study outcomes is to detect the questions that are beyond students’ abilities so that the automated system can give them additional instructions. The potential application of the question-independent study outcomes is assisting the correctness rate in the evaluating problems’ difficulty levels.

## 2. Related Work

Eye-tracking technology has been used in studying cognitive states during the problem-solving process in many previous works, including [12], IQ [13], cognitive states [9,14,15], visual perception [16], and visual cognition [17]. Beyond learning science, eye-tracking technology also performs well in safe driving assessment [18], diagnosis of Alzheimer’s disease [19], recognizing stimuli types [20,21], and gaming [22]. These studies concluded that a lot of eye-tracking characteristics, such as fixation duration [23], saccades, path distance, blink rate, and pupil diameter, are considered to reflect important information of the cognitive process. The existing widely accepted standards for analyzing eye-movement characteristics are well-documented in [24], and useful open-source tool for eye-tracking data analysis can be found in [25]. In recent years, several works suggested the possibility to build a proactive tutoring system based on eye-tracking technology [9,26,27], but their studies did not involve widely used question forms, such as multiple-choice questions. To build a practical proactive tutoring system, it must be able to extract useful information and build predictive models from eye-tracking data collected in normal e-learning scenarios instead of well-designed experiments. However, most of the previous works focused on building predictive models or drawing important information from their specific experiments which could not be adapted into practical usage.

Other than the adaptation of models, one requirement of a good proactive tutoring system is unobtrusiveness. Before eye-tracking technology was applied widely, research used the thinking aloud protocol [28,29]—i.e., speaking out what is on one’s mind during the thinking process—to probe the cognitive process. However, this method interferes with the participants’ behavior because it requires participants to perform extra tasks beyond the regular thinking process [30]. By contrast, eye-tracking technology has little influence on the participants’ cognitive process. Glockner [11] indicated that eye-tracking technology has less possibility to alter the decision process. However, it is rarely possible to completely replace verbal information with eye-tracking data to investigate human cognitive processes because of the non-immediacy of eye-tracking data (i.e., no effective information can be obtained directly from pure eye-tracking data). While it is possible to use eye-tracking individually to build a tutoring system because of the information contained in answers, drafts and question descriptions are sufficient to provide sideshows of the problem-solving process.

The information of both the stimuli and the problem solvers could be reflected in the eye-tracking data. The types of stimuli have been successfully predicted by using eye-tracking data in reading materials types [21] and landscape types [20]. In addition, several former works investigated the eye-movement patterns in the problem-solving process and connected them to other information related to the participants. Yarbus [2] first suggested that human eye-movement pattern is influenced by different task demands. To further investigate the controversial hypothesis that it is possible to decode the observer’s task from eye movement [31,32], Borji et al. [33] reanalyzed Greene et al.’s data and collected new data from scenes expanded from Yarbus’ study [2]. By building k-nearest-neighbor and boosting models, the results show small but significant above chance accuracy levels. Based on that, they concluded that task decoding from eye-tracking data is possible. Eivazi and Bednarik [9] modeled human cognitive states using eye-tracking technology as well as the thinking aloud protocol. Their work concluded that eye-tracking data contains important information about cognitive states and could potentially be used to build predictive models for performance and mental behaviors in the problem-solving process. Their real-time model of cognitive states can help proactive systems understand users’ learning conditions and, more importantly, the models adapt well to unseen samples. Simola et al. [14] studied how cognitive states alternate during information search tasks. They divided the tasks into three steps (word searching, finding an answer, and choosing the most interesting title from a list) and trained a Hidden Markov Model (HMM) to predict the current task type out of three types. Their classifier achieved an accuracy of 60.2%. Their study shows that eye-tracking data contains the necessary information for determining task type. More recently, a generative framework, based on HMM, is proposed for investigating multiple moving objects using eye-tracking data [34]. In addition to responding to the problem-solving process, eye-movement data is also related to the ability of the subject. In [13], Hayes et al. presented a novel method to process sequential eye-tracking data by using successor representation on Raven’s advanced progressive matrices (APM). They pointed out the limitations of other methods such as transition probability matrices and Markov models [14,35] and gained unprecedented accuracy to predict APM scores. Mavrikis and Chen et, al. discussed the relationship between eye-movement patterns and GPA [4,26]. In [26], Mavrikis focused on eye-tracking data when solving online learning problems. They managed to improve instructional design to facilitate online learning based on recorded eye movement. Furthermore, Chen et al.’s work [4] showed a relationship between eye-movement patterns in spatial visualization and science grades. Prior knowledge [36] and performance [37,38] of problem solvers is also reflected in the eye-movement pattern.

## 3. Experimental Procedure

In this section, we illustrate the data we obtained and how we processed it. The collected data is divided into two parts: (1) eye-tracking data collected from participants during their spatially related problem-solving process (Study 1), and (2) participants’ feedback on the difficulty of the questions (Study 2). Study 2 plays an auxiliary role to help us assess the difficulty of materials used in Study 1.

### 3.1. Eye-Tracking Data

To investigate human cognition states and eye movement, we chose spatial visualization problems in the form of multiple-choice questions as our target example since (1) multiple-choice question is one of the most commonly used form of e-learning presently and (2) Chen et al. [4] claimed that spatial problems involve a lot of mental deduction process which could be reflected in participants’ eye movement. The eye-tracking data we used in this study (Study 1) was collected with first-year engineering students enrolled in an Introduction to Spatial Visualization course at Virginia Tech. Participants were invited to participate in the study as an out of class activity. The study participation was voluntary, and no course credit was given to those who chose to participate.

#### 3.1.1. Participants

This study recruited 88 students, with a 1:1 gender ratio. All participants were first-year engineering first-year students. Participants self-reported normal or corrected-to-normal vision. The study was conducted in an isolated room with minimal distractions. The participant was seated in an adjustable chair. A chinrest was used to ensure the quality of eye-tracking. The distance between the chinrest and the laptop screen was approximately 60 cm, of which minor adjustment by the participants was permitted. The computer collecting the data was a Dell laptop with an Intel Core i7 processor.

The Mangold Vision Eye-Tracker package was used for data collection, experiment design, and data analysis. The VT3 mini eye-tracker has an accuracy of approximately 0.5 visual angle and uses dark pupil binocular or monocular tracking.

#### 3.1.2. Measures

Problems included four questions from the “Purdue Spatial Visualization Test: Rotation” (PSVT: R) and six questions from the Santa Barbara Solids Test (SBST). The PSVT: R problems test the ability to perform three-dimensional mental rotations using two-dimensional perspectives. Participants were shown an object (A) in its original position and rotated position, then they are shown a different object (B) and are asked to find object B’s rotated shape if that same rotation is applied to it [39]. The SBST problems test the ability to identify the two-dimensional cross sections of a three-dimensional geometric solid (again, in a two-dimensional perspective) [40]. Participants are shown an object intersected by a cutting plane and they must identify the two-dimensional intersection.

#### 3.1.3. Procedure

Upon arrival at the experiment location, each participant was given an IRB consent form and was asked to read it carefully. Once the participant signed the informed consent form, the participant was invited to be seated in the isolated room. Then, the participant was asked to place their chin on the chinrest and adjust the chinrest to a comfortable height. When the participant felt comfortable with the position, the experimenter began eye-tracker calibration until the accuracy was above 90%. Three slides of instructions were presented to the participant on the PSVT: R and SBST tests. The participants were given the opportunity to ask questions before the experimenter exited the room and observed through a one-way mirror when the experiment began.

This experiment included 4 PSVT: R and 6 SBST tasks. The order of the tasks was randomized to counterbalance potential learning effects and participants were randomly assigned to each trial. For the PSVT: R, the tasks for each difficulty were chosen based on previous student performance; the SBST was chosen based on the complexity of the task as there is no performance data to draw upon.

For each task, the participant was given 20 seconds to answer. The participant could proceed to the next task before the time limit expires. Before each task, a focus slide appeared for five seconds. The focus slide ensured a baseline position of starting fixation and focus on the upcoming task. The participants were asked to write down their answers for each task to ensure engagement; however, the answers were not used in the analysis.

**Figure 1:**
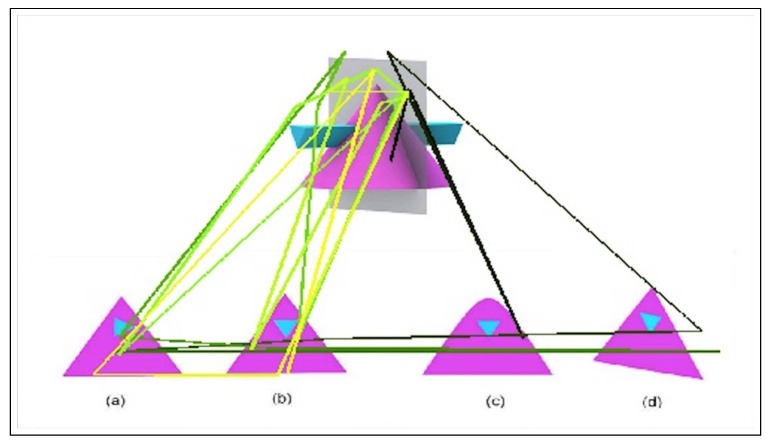
An example of the eye-movement patterns during spatial problem-solving. The path is represented by a line that gets brighter in color (black-green-yellow) as time progresses. Each answer option (**a**–**d**) represents an option for how the cross section of the gray plane with the 3D object could look like.

An example from the used dataset for SBST problems is shown in Figure 1 along with the eye fixation path that we generated from the eye-tracking data. The dataset has records of six features and three of them are used in this work; the ones used are x-position, y-position, and the time gap between two eye movements.

### 3.2. Difficulty Level

To investigate the difficulty of PSVT: R and SBST problems, we conducted another user study (Study 2) to probe difficulty differences. Data was collected on 23 students, aged between 20 and 22, solving 10 questions that included the same four PSVT: R and six SBST used in the first study (described above). All participants majored in engineering and had limited training in spatial visualization. The problems were presented on the computer screen of size 13.75 × 9.48 inches and resolution 2880 × 1800 and participants had up to 20 seconds to answer each question (they can skip to the next question if they are ready before the 20 seconds elapse). The time limitation of 20 seconds aims to meet the experiment condition of the Virginia Tech study which also conducted a time-limited test with up to 20 seconds for each question. When they finished with all the questions, they had a chance to review each question for 10 seconds, then they gave their ranking of the mental effort involved in solving each question and grouped the questions into difficulty levels. The ranking and grouping were done separately for PSVT: R and SBST questions.

The PSVT: R were grouped into HARD and EASY by Chen et al. (refer to [4]), based on a pilot test, and SBST questions were also grouped based on an intuitive feeling of complexity in Study 1. However, in our user study, participants were more likely to group SBST questions into three difficulty levels rather than the two levels. Therefore, we encouraged participants to group PSVT: R into two levels and group SBST into three levels in their difficulty ranking process.

### 3.3. Approaches

To investigate the eye-tracking data of multiple-choice questions, we performed our work in two steps. First, in the feature extraction, like many works that studied eye-movement data, we divided the area of eye-tracking data according to practical significance. The division method will be presented in Section 3.4. Second, we conducted two kinds of tests to explore the possibility to predict difficulty levels from eye-tracking data. In each test, machine learning models were constructed to perform question-dependent and question-independent classification tasks.

### 3.4. Feature Extraction

**Figure 2:**
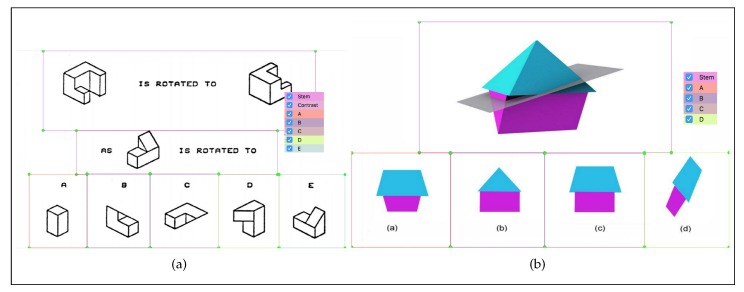
Example divisions of the time-limited experiment for (**a**) PSVT: R division and (**b**) SBST division.

Different AOIs of the questions have different information that contributed to the mental deduction process. The difference leads to eye movement changing when going through an AOI to another [4]. To obtain useful information from questions, each question in the experiment was divided into seven (A, B, C, D, E, Stem, Contrast) and five (A, B, C, D, Stem) different areas for PSVT: R and SBST questions respectively. Each area contains important information which is essential to solve the problem. All areas belong to two categories: QUESTION, which includes Stem and Contrast, and OPTION, which includes A, B, C, D, E. Figure 2 shows the division method in which participants learn the description of tasks in QUESTION and match their answer with options in OPTION. We grouped AOIs in the above way because options and question descriptions are essential parts of multiple-choice questions. By grouping in that manner, we could ensure the practical value of this work.

Based on the fixation data, we computed several features and found seven of them—shown in Table 1—that show a significant impact on classifying the difficulty level of time-limited tasks. Although the eye-tracker has been calibrated before the user study, it is not accurate enough to let all fixation located in the original position. After checking data manually, outliers were removed. In the rest of the data, only a few fixation points are out of all the boxes and, in this work, they are labeled as BLANK and will not be considered in our classification procedure.

We chose features in Table 1 due to three reasons. First, Velichkovsky et al. [10] and Chen et al. [4] stated that fixation duration in different AOI contains important information about difficulty of problems. Second, the duration length was simply determined as 10 seconds because it was half of the maximum solving time. Third, since multiple-choice questions have fixed locations of options and stem, features such as path distance and saccade angle are meaningless. Considering the eye-tracking data was collected from a time-limited test, although participants’ habits vary a lot, they should follow a general sequence of learning behaviors. For example, going through the question description first. Based on that, fixation duration implies information about the order in which questions are answered. That is why we divided solving time into several small periods to extract time-different features and assume fixation duration in each AOI as our targeted measure. To better illustrate the eye-tracking characteristics, some analyses of extracted features are presented in what follows.

**Figure 3:**
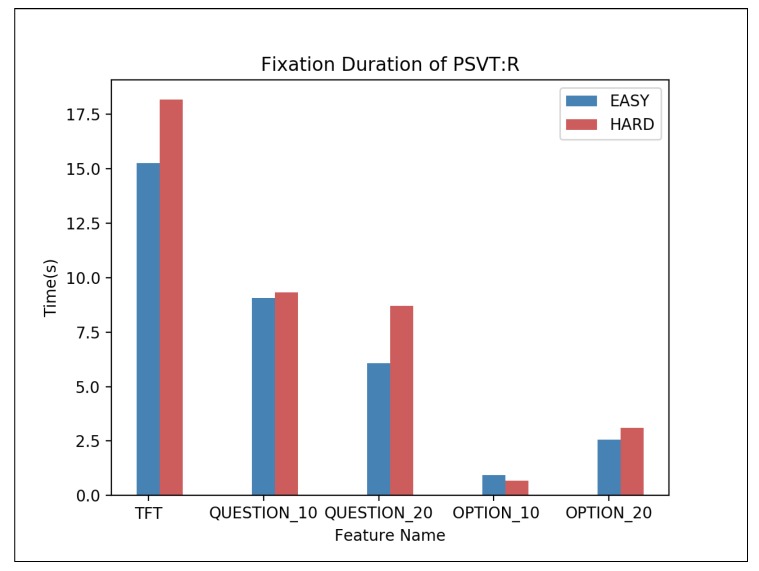
Average fixation duration of PSVT: R—one-way ANOVA results: TST (F = 27.771, p = 0.00 **), QUESTION_10 (F = 21.759, p = 0.00 **), QUESTION_20 (F = 13.949, p = 0.00 **), OPTION_10 (F = 0.672, p = 0.570), OPTION_20 (F = 16.283, p = 0.00 **), ** p < 0.01.

To learn useful information of multiple-choice questions, the average fixation duration of PSVT: R is shown in Figure 3. For both HARD and EASY, fixation duration in QUESTION decreases with time and fixation duration in OPTION increases with time. For TST and fixation duration in QUESTION, HARD problems have a longer time of them than EASY. Since the time spent gazing at BLANK is not considered in TST, the sum of the time spent observing the QUESTION and the OPTION is not necessarily equal to the TST.

**Figure 4:**
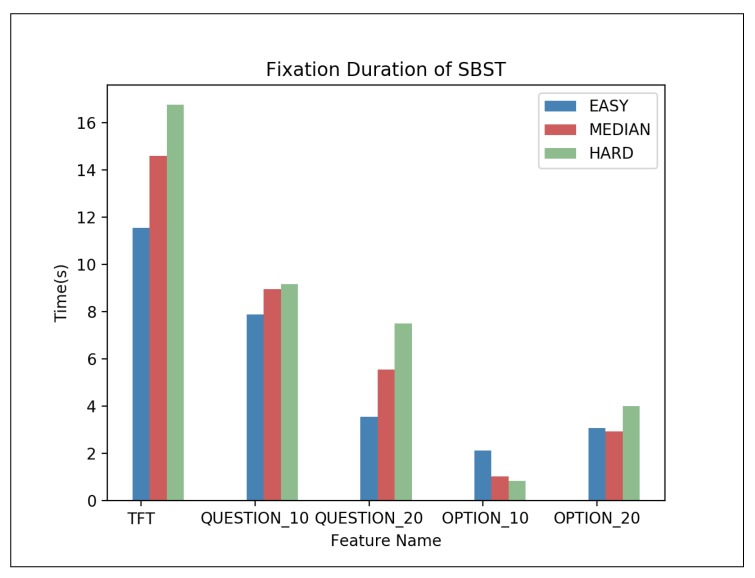
Average fixation duration of SBST—one-way ANOVA results: TST (F = 10.044, p = 0.00 **), QUESTION_10 (F = 39.303, p = 0.00 **), QUESTION_20 (F = 13.067, p = 0.00 **), OPTION_10 (F = 15.695, p = 0.00 **), OPTION_20 (F = 31.502, p = 0.00 **), ** p < 0.01.

Average fixation duration of SBST is shown in Figure 4. Like PSVT: R, more difficult problems tend to have longer TST. However, for fixation duration in each AOI, the distribution of SBST is more variable, but we can still find that the fixation duration in QUESTION and OPTION decreases with time which contrary to the trend from PSVT: R. This reflects that for different types of questions, even if the questions all belong to the spatial visualization problems, people’s eye-movement habits are quite different. Based on that, we can safely speculate that for different types of questions, people tend to have different eye-movement patterns to solve questions from different difficulties. That means it will be difficult to train a general model to deal with all types of questions, which results in the complexity of using eye-tracking data to reflect the difficulty of the problems.

## 4. Results and Discussion

In this section, first, we present results of user study for ranking and grouping questions into several difficulty levels. Second, we show the performance of our predictive models and illustrate how they could be applied to a proactive tutor system. Our predictive tasks contain two steps. We first aim to build models to classify difficulty level with labeled data (Question-Dependent), then we try to adapt our predictive models to unseen but similar questions to the model and evaluate the performance of them (Question-Independent). 10-fold cross-validation was applied for all our model evaluation processes [41].

### 4.1. Difficulty Ranking

The user study results are shown in Table 2. In Section 3.2, we have shown how we reached the difficulty level and assign a difficulty level to each question based on the most voted level. According to that, we conclude from the result that PSVT: R questions could be classified into EASY (includes PSVT: R 1 and PSVT: R 2) and HARD (includes PSVT: R 3 and PSVT: R 4); SBST questions could be classified into EASY (includes SBST 1), MEDIUM (includes SBST 2, SBST 3 and SBST 5), and HARD (includes SBST 4 and SBST 6).

**Figure 5:**
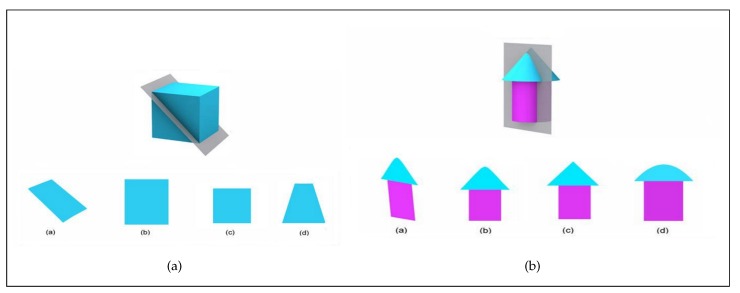
A question reported confusing in our user study—(**a**) SBST 2 and (**b**) SBST 3.

Figure 5 shows two SBST questions which were reported confusing. For SBST 2, some of the participants reported they could not discriminate between option B (rectangle) and C (square). This made them hesitate for a long time or randomly select a potential option. SBST 3 has a higher difficulty ranking than SBST 2. The complexity of this question is from the cross-section location. When participants reviewed SBST 3 again to rank the difficulty, some of them reached a different answer. They reported they did not realize that the cross-section did not pass through the conical busbar for the first time. All participants who did not be aware of the correct cross-section location grouped SBST 3 with EASY. These confusing parts have a big influence on the eye-movement pattern and result in different classification result which we will discuss later.

### 4.2. Features and Classifier Selection

To find out the suitable classifier and features, multiple models have been created. As many previous works [24,37,42], random forest (RF), decision tree (DT), Naïve Bayes (NB), Logistic Regression (LR), and Support Vector Machine (SVM) performed well in decoding and reasoning tasks with eye-tracking data. Other than classifier types, features also play an important role with respect to the performance of assigned classification tasks. We assumed time-different features could have better performance in our classification tasks. To validate that, we grouped two sets of features with different duration length. Feature group A which includes TST, QUESTION_10, QUESTION_20, OPTION_10, and OPTION_20; and feature group B which includes TST, QUESTION, and OPTION. We then trained and evaluated every combination of five classifiers and the two feature groups.

Table 3 presents the average accuracy and standard deviation—in parentheses—of ten-fold cross-validation of all combinations of classifiers and feature groups. The label of the results of Table 3 is determined by the difficulty levels referring to difficulty ranking results. SVM and LR outperform other classifiers. Since our original intention is investigating the relationship between eye-movement pattern and difficulty level, like other eye-tracking papers, we only chose one classifier (SVM) as our targeted classifier to analyze and conduct further experiments. In [42], SVMs are considered to have three characteristics suitable for human cognitive state classification. First, it is impossible to express a human cognitive state in a linear model, and SVMs can efficiently calculate nonlinear models just like linear models. Second, SVMs can be used when prior knowledge of the dataset is unavailable. Third, SVMs minimize the upper bound of the generalization error, which enables SVMs to generate a more robust model. Consequently, with SVM classifier, models trained with feature group A have a higher accuracies than those trained with feature group B, but the standard deviation of feature group A models are approximately twice as much as that of group B. However, the standard deviation of feature group A models remains in the acceptable range, so we chose the features in feature group A as our features for the remainder of this paper.

### 4.3. Question-Dependent Test

In this section, we illustrate our approach to classify samples into their corresponding difficulty levels. Two kinds of labels were involved in classification tasks. The first is the ’Ungrouped’, which means that the sample label was assigned as its corresponding question index. For example, the first question in SBST will be labeled as SBST 1. The second is the ’Grouped’, which means that all samples were labeled by difficulty levels referring to the results of Study 2. This way, grouped SBST data and ungrouped SBST data have 3 and 6 classes, respectively. For PSVT: R data, the number of classes is 2 and 4, respectively. We employed a grid search in our training process to gain better results. All data involved is normalized from zero to one, as shown Equation (1) where *X* is a data sample.

**Figure 6:**
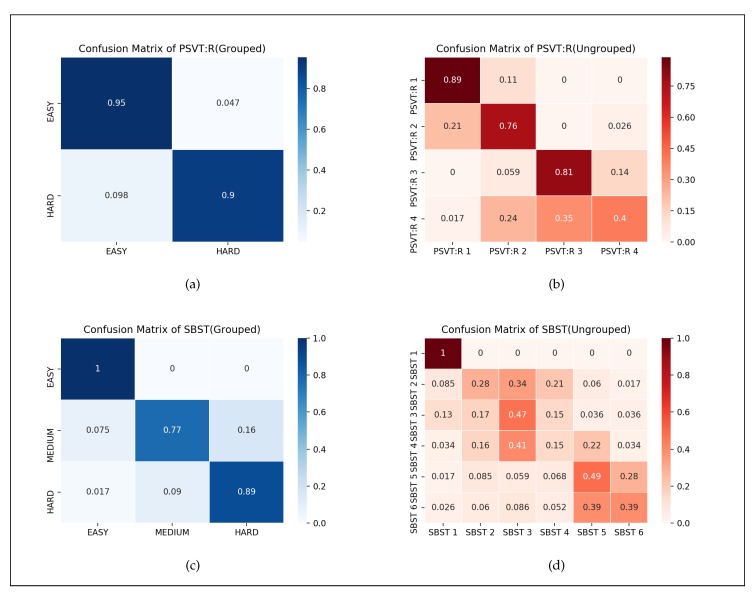
Confusion matrix of question-dependent tasks of SBST & PSVT: R—(**a**) SBST (Grouped); (**b**) SBST (Ungrouped); (**c**) PSVT: R (Grouped); (**d**) PSVT: R (Ungrouped).

To further investigate the ability of the SVM, we calculate the confusion matrix of ’Grouped’ and ’Ungrouped’ data. Figure 6b,d show the confusion matrix of ungrouped SBST and PSVT: R questions. From the confusion matrix, we can easily observe boundaries among classes are blurring. For example, SBST 2, SBST 3 and SBST 4 could not be discriminated between each other by the classifier. Compared it to our difficulty levels, we found the classifier has the weak capability to correctly classify samples belonging to the same difficulty level. In ungrouped PSVT: R samples, PSVT: R 4 has the lowest accuracy and are partly classified into PSVT: R 2 and PSVT: R 3. This can be explained by participants’ behavior in the user study. Because of the extraordinary complexity of PSVT: R 4, most of the participants did not get the correct answer. In the subsequent talk, many students reported that they skipped the question directly because of the lack of ideas. The direct skip leads to a completely different eye-tracking pattern and then results in misclassification.

Figure 6a,c show the confusion matrices of classification results of grouped data. The results of both SBST and PSVT: R samples are on a precise basis. As the results show, when the SBST questions are labeled by three difficulty levels, the classifier can effectively classify them through eye-tracking data. Also, the classifier with grouped PSVT: R samples have an accuracy of more than 90%. The successful classification of grouped data inspires us to continue to explore the prediction of eye-tracking data in terms of difficulty level of tasks. It can be inferred from our classification results that the eye-movement data of the same difficulty level problem has similar characteristics and can, therefore, be classified by the classifier. Although, as mentioned in Section 3.4, eye-movement patterns of different types of questions vary a lot, predicting difficulty level of homogeneous questions but unseen to the classifier should be possible.

(1) $$X^{\prime }=\frac{X-X_{\min}}{X_{\max}-X_{\min}}$$

### 4.4. Question-Independent Test

To extend the usability of the classification model, we conducted the question-independent classification tasks and analyzed the performance based on the existing experimental data. For PSVT: R questions, we assumed one of four questions as testing data and the other three as training data. For SBST questions, since only one question is at EASY level, it could not be tested since no other questions on the same level, so we trained five models to test the other five questions. The label in the subsequent analysis should be difficulty levels defined in Section 3.2. Considering the imbalance of the sample number for each class, the class weights have been balanced when training the classifier.

Table 4 shows the results of the question-independent test of SBST questions. The average accuracy of this question-independent test is 66.2% (33% randomly). Because of the small sample volume, the robustness of our models may have room for improvement. However, because it is significantly above chance levels, it is enough to show that our classifier has the capability to classify samples into the correct difficulty level. As per our difficulty ranking, shown in Table 2, SBST 2 and SBST 5 are the easiest and hardest questions respectively among SBST questions. The accuracies of SBST 2 and SBST 5 are relatively higher than others, which indicates that classifiers may have better performance in dealing with eye-tracking data of questions with obvious difficulty difference. There is also another reason that could have a high influence on classification results: when we conducted the user study, two SBST questions were reported confusing, which led to apparent eye-tracking change. This change confuses the classifier and resulted in low accuracy.

We conducted classification tasks separately for the four PSVT: R questions. Table 5 shows that the results of question-independent tasks of PSVT: R have an average accuracy of 79.51%. Two reasons may contribute to a higher accuracy rate than SBST models. First, there are only two difficulty groups in PSVT: R questions and none of them were reported confusing. Since confusing questions block students‘ problem-solving processes, eye-tracking data could further reflect students’ inner thinking if students could analyze questions step by step without being confused. Secondly, four PSVT: R questions are in a strictly homogeneous type of problem which needs participants to conduct very similar works for each question. However, for SBST questions, although they belong to cutting problems, they need participants to discover different aspects of spatial features. The results suggest that our predict model works better when the input questions belong to strictly the same type.

As we worked on the model predictions, we found that samples classified into EASY are more likely to get a correct answer even in the situation of HARD questions but an EASY eye-movement pattern. We speculate that the eye-movement pattern also reflects the confidence of the user during the problem-solving process. Due to the limitation of our dataset, we could not further explore it in this paper. However, if this trend could be shown to be significant, it would also contribute to building a proactive tutoring system by providing information about students’ performance.

## 5. Conclusions and Future Work

In this paper, we studied the possibility of using eye-movement data to predict the difficulty of the problem and built SVM-based models to verify it. We first analyzed the pattern of eye-movement data when users solve problems of different difficulty levels. From the differences in eye movements of difficulties reflected in the results, we proposed using SVMs to predict the difficulty level of the questions. Two kinds of models were built to classify and predict the difficulty level of spatial visualization problems. One is to classify difficulty levels of the questions corresponding to the data trained by the classifier. The other is to predict the difficulty of the new questions. Our ultimate goal is to make the computer understand the user’s problem-solving process through the analysis of eye-movement data, so as to dynamically adjust the difficulty level of the problem and guide the user. Our findings show that even if the topic types are not accurately the same, the classifier can still have an average accuracy of 87.60% for the known questions, and 72.87% for the unknown questions. This may give researchers and instructors who are committed to the problem-solving process another way to evaluate and improve the performance of problem-solving.

Two limitations of our experiment would be solved in the future. First, to fit the most common type of e-learning, we choose eye-tracking data recorded from solving processes of multiple-choice questions. The questions test students’ spatial visualization ability and require participants to conduct spatial imagination in their mind. Our findings showed that eye-tracking data could be used to classify difficulty levels of spatial visualization problems; however, other types of problems may have negative impact on accuracy such as questions involving little mental thinking but demand pure calculations. To classify other types of problems, eye-tracking may not be sufficient and other supplementary materials may be needed, such as a draft of the calculation process. Moreover, since the number of our stimuli is not large, the robustness may not be ideal. Considering our results are significantly above the chance levels, we can still safely conclude that eye-tracking data could be used to predict difficulty levels. We plan to expand the stimuli set in upcoming studies.

Future steps of this research include investigating if collecting more eye-tracking data could improve the performance and robustness of current models, conducting experiments to investigate if multiple-choice questions of other materials allow the classifier to classify difficulty levels of tasks from eye-tracking data, and developing real-time proactive tutoring systems that use eye-tracking to understand users’ mental activity. The long-term goal of our study is to build intelligent systems that could follow the users’ learning processes and provide proactive guidance for better and more efficient learning.

## Figures and Tables

**Table 1:** Features of eye movement.

Features	Description
Total solving time(TST)	Total time of answering task
Fixation duration in QUESTION in first 10 seconds (QUESTION_10)	Sum of fixation duration in QUESTION in first 10 seconds
Fixation duration in OPTION in first 10 seconds (OPTION_10)	Sum of fixation duration in OPTION in first 10 seconds
Fixation duration in QUESTION in second 10 seconds (QUESTION_20)	Sum of fixation duration in QUESTION in second 10 seconds
Fixation duration in OPTION in second 10 seconds (OPTION_20)	Sum of fixation duration in OPTION in second 10 seconds
Fixation duration in QUESTION (QUESTION)	Sum of fixation duration in QUESTION
Fixation duration in OPTION (OPTION)	Sum of fixation duration in OPTION

**Table 2:** User study results. Please note that the ranking score is the average ranking order of questions.

Questions	Ranking Score	Correctness Rate	Easy	Medium	Hard
PSVT: R 1	1.04	91.3%	23	N/A	0
PSVT: R 2	2.09	82.6%	23	N/A	0
PSVT: R 3	3.17	43.5%	0	N/A	23
PSVT: R 4	3.70	30.4%	0	N/A	23
SBST 1	1.00	100%	23	0	0
SBST 2	2.00	91.3%	10	13	0
SBST 3	3.04	69.6%	5	18	0
SBST 4	4.00	95.6%	0	23	0
SBST 5	5.52	39.1%	0	0	23
SBST 6	5.43	52.2%	0	0	23

**Table 3:** Results of the question-dependent test.

Method	PSVT: R		SBST	
	Feature Group A	Feature Group B	Feature Group A	Feature Group B
RF	0.71 (±0.19)	0.68 (±0.09)	0.77 (±0.26)	0.61 (±0.01)
DT	0.73 (±0.26)	0.62 (±0.04)	0.83 (±0.27)	0.55 (±0.04)
NB	0.67 (±0.11)	0.65 (±0.10)	0.66 (±0.30)	0.55 (±0.28)
LR	0.93 (±0.31)	0.68 (±0.13)	0.84 (±0.24)	0.61 (±0.08)
SVM	0.93 (±0.26)	0.70 (±0.12)	0.84 (±0.23)	0.62 (±0.10)

**Table 4:** Results of SBST.

Testing Question	Accuracy	Training Questions
SBST 2	71.79%	SBST 1, SBST 3, SBST 4, SBST 5, SBST 6
SBST 3	52.67%	SBST 1, SBST 2, SBST 4, SBST 5, SBST 6
SBST 4	62.06%	SBST 1, SBST 2, SBST 3, SBST 5, SBST 6
SBST 5	79.66%	SBST 1, SBST 2, SBST 3, SBST 4, SBST 6
SBST 6	64.95%	SBST 1, SBST 2, SBST 3, SBST 4, SBST 5

**Table 5:** Results of PSVT: R.

Test Question	Accuracy	Training Questions
PSVT: R 1	76.92%	PSVT: R 2, PSVT: R 3, PSVT: R 4
PSVT: R 2	70.94%	PSVT: R 1, PSVT: R 3, PSVT: R 4
PSVT: R 3	88.13%	PSVT: R 1, PSVT: R 2, PSVT: R 4
PSVT: R 4	82.05%	PSVT: R 1, PSVT: R 2, PSVT: R 3

## References

[1] Blikstein P., Worsley M. (2016). Multimodal learning analytics and education data mining: Using computational technologies to measure complex learning tasks. J. Learn. Anal..

[2] Yarbus A. (1967). Eye Movements and Vision.

[3] Rayner K. (1998). Eye movements in reading and information processing: 20 years of research. Psychol. Bull..

[4] Chen Y.C., Yang F.Y. (2014). Probing the relationship between process of spatial problems solving and science learning: An eye tracking approach. Int. J. Sci. Math. Educ..

[5] Just M.A., Carpenter P.A. (1976). Eye fixations and cognitive processes. Cognit. Psychol..

[6] Roach V.A., Fraser G.M., Kryklywy J.H., Mitchell D.G., Wilson T.D. (2017). Time limits in testing: An analysis of eye movements and visual attention in spatial problem solving. Anatom. Sci. Educ..

[7] Kaller C.P., Rahm B., Bolkenius K., Unterrainer J.M. (2009). Eye movements and visuospatial problem solving: Identifying separable phases of complex cognition. Psychophysiology.

[8] Conati C., Merten C. (2007). Eye-tracking for user modeling in exploratory learning environments: An empirical evaluation. Knowl. Based Syst..

[9] Eivazi S., Bednarik R. Predicting Problem-Solving Behavior and Performance Levels from Visual Attention Data. Proceedings of the Workshop on Eye Gaze in Intelligent Human Machine Interaction at IUI.

[10] Velichkovsky B.M., Rothert A., Kopf M., Dornhöfer S.M., Joos M. (2002). Towards an express-diagnostics for level of processing and hazard perception. Transp. Res. Part F Traffic Psychol. Behav..

[11] Glöckner A., Herbold A.K. (2011). An eye-tracking study on information processing in risky decisions: Evidence for compensatory strategies based on automatic processes. J. Behav. Decis. Mak..

[12] Chen S., Epps J. (2014). Using task-induced pupil diameter and blink rate to infer cognitive load. Hum. Comput. Interact..

[13] Hayes T.R., Petrov A.A., Sederberg P.B. (2011). A novel method for analyzing sequential eye movements reveals strategic influence on raven’s advanced progressive matrices. J. Vis..

[14] Simola J., Salojärvi J., Kojo I. (2008). Using hidden Markov model to uncover processing states from eye movements in information search tasks. Cognit. Syst. Res..

[15] Nisiforou E.A., Laghos A. (2013). Do the eyes have it? Using eye tracking to assess students cognitive dimensions. Educ. Media Int..

[16] Gegenfurtner A., Lehtinen E., Säljö R. (2013). Expertise Differences in the Comprehension of Visualizations: A Meta-Analysis of Eye-Tracking Research in Professional Domains. Educ. Psychol. Rev..

[17] Rayner K., Loschky L.C., Reingold E.M. (2014). Eye movements in visual cognition: The contributions of George W. McConkie. Vis. Cognit..

[18] Palinko O., Kun A.L., Shyrokov A., Heeman P.A. Estimating Cognitive Load Using Remote Eye Tracking in a Driving Simulator. Proceedings of the Symposium on Eye-Tracking Research & Applications (ETRA).

[19] Fernández G., Castro L.R., Schumacher M., Agamennoni O.E. (2015). Diagnosis of mild Alzheimer disease through the analysis of eye movements during reading. J. Integr. Neurosci..

[20] Król M.E., Król M. (2020). The right look for the job: Decoding cognitive processes involved in the task from spatial eye-movement patterns. Psychol. Res..

[21] Kai K., Utsumi Y., Shiga Y., Kise K., Bulling A. I Know What You are Reading: Recognition of Document Types Using Mobile Eye Tracking. Proceedings of the International Symposium on Wearable Computers.

[22] Smith J.D., Graham T. Use of Eye Movements for Video Game Control. Proceedings of the ACM SIGCHI International Conference on Advances in Computer Entertainment Technology.

[23] Vansteenkiste P., Cardon G., Philippaerts R., Lenoir M. (2015). Measuring dwell time percentage from head-mounted eye-tracking data–comparison of a frame-by-frame and a fixation-by-fixation analysis. Ergonomics.

[24] Duchowski A.T. (2017). Eye Movement Analysis.

[25] Dink J.W., Ferguson B. Eyetracking R: An R Library for Eye-Tracking Data Analysis. www.eyetracking-r.com.

[26] The B., Mavrikis M. A Study on Eye Fixation Patterns of Students in Higher Education using an Online Learning System. Proceedings of the Sixth International Conference on Learning Analytics & Knowledge.

[27] Król M., Król M. (2019). Learning from peers’ eye movements in the absence of expert guidance: A proof of concept using laboratory stock trading, eye tracking, and machine learning. Cognit. Sci..

[28] Gerjets P., Kammerer Y., Werner B. (2011). Measuring spontaneous and instructed evaluation processes during Web search: Integrating concurrent thinking-aloud protocols and eye-tracking data. Learn. Instruct..

[29] Elling S., Lentz L., De Jong M. (2012). Combining concurrent think-aloud protocols and eye-tracking observations: An analysis of verbalizations and silences. IEEE Trans. Prof. Commun..

[30] McCrudden M.T., Magliano J.P., Schraw G. (2011). The effect of diagrams on online reading processes and memory. Discourse Process..

[31] Henderson J.M., Shinkareva S.V., Wang J., Luke S.G., Olejarczyk J. (2013). Predicting cognitive state from eye movements. PLoS ONE.

[32] Greene M.R., Liu T., Wolfe J.M. (2012). Reconsidering Yarbus: A failure to predict observers’ task from eye movement patterns. Vis. Res..

[33] Borji A., Itti L. (2014). Defending yarbus: Eye movements reveal observers’ task. J. Vis..

[34] Kim J., Singh S., Thiessen E.D., Fisher A.V. (2020). A hidden Markov model for analyzing eye-tracking of moving objects. Behavior Research Methods.

[35] Jansen A.R., Marriott K., Yelland G.W. (2007). Parsing of algebraic expressions by experienced users of mathematics. Eur. J. Cognit. Psychol..

[36] Hinze S.R., Rapp D.N., Williamson V.M., Shultz M.J., Deslongchamps G., Williamson K.C. (2013). Beyond ball-and-stick: Students’ processing of novel STEM visualizations. Learn. Instruct..

[37] Nüssli M.A., Jermann P., Sangin M., Dillenbourg P. Collaboration and Abstract Representations: Towards Predictive Models based on Raw Speech and Eye-Tracking Data. Proceedings of the 9th International Conference on Computer Supported Collaborative Learning.

[38] Hu Y., Wu B., Gu X. (2017). An eye tracking study of high-and low-performing students in solving interactive and analytical problems. J. Educ. Technol. Soc..

[39] Bodner G.M., Guay R.B. (1997). The Purdue visualization of rotations test. Chem. Educ..

[40] Cohen C.A., Hegarty M. (2012). Inferring cross sections of 3D objects: A new spatial thinking test. Learn. Individ. Differ..

[41] Hsu C.W., Chang C.C., Lin C.J. A Practical Guide to Support Vector Classification. https://www.csie.ntu.edu.tw/~cjlin/papers/guide/guide.pdf.

[42] Liang Y., Reyes M.L., Lee J.D. (2007). Real-time detection of driver cognitive distraction using support vector machines. IEEE Trans. Intell. Transp. Syst..

